# Random Periareolar Fine-Needle Aspiration: The New Pap Smear of the Breast?

**DOI:** 10.6004/jadpro.2012.3.6.9

**Published:** 2012-11-01

**Authors:** Joanne Lester, Lisa Diane Yee

**Affiliations:** From The Ohio State University Arthur G. James Cancer Hospital & Richard J. Solove Research Institute, Columbus, Ohio

## Abstract

Random periareolar fine-needle aspiration continues to gain scientific credence in the short-term identification of women at increased risk for breast cancer. As this technique becomes more widely used, APs may seek to be trained in an effort to expand clinical trials, and someday provide a "Pap smear of the breast" for the women who need it most.

Women at increased risk of developing breast cancer rely on clinical examination, radiographic studies (e.g., mammogram, ultrasound, and MRI), biopsy, and genetic tests to predict their personal risk of breast cancer. Providers also use information such as family history of breast or ovarian cancer, age at which a relative’s (or one’s own) diagnosis occurred, age at menarche/menopause, and age at first birth (Cyr et al., 2011). These factors may identify women at increased risk, though an individual factor or cumulative factors cannot accurately predict the absolute risk or occurrence of breast cancer.

Preventative measures for breast cancer include restriction of exogenous hormones, removal of endogenous hormone sources (e.g., bilateral oophorectomy), administration of selective estrogen receptor modulators (e.g., tamoxifen or raloxifene), and prophylactic bilateral mastectomy. When any of these intervention(s) are chosen, it remains unknown whether the individual’s chosen intervention(s) will be significant and effective for her over time. It is certain that some women at high risk take no action and do not develop breast cancer, while other women "do it all" and still ultimately develop a local or metastatic breast tumor. Notwithstanding, most breast cancer risk reduction interventions are associated with negative side effects (Hoffman, Pellenberg, Ibarra-Drendall, & Seewaldt, 2012) and the potential of long-term symptoms.

Practitioners need minimally invasive techniques that can effectively identify the biologic origins of breast cancer and provide a reasonable and reliable approach to surveillance for breast cancer development. Ideally, the biomarkers would enable short-term prediction of breast cancer development, measure the effect of interventions, demonstrate reliability and validity, and ultimately prevent the development of invasive breast cancer. Surrogate biomarkers for breast cancer might include breast tissue, serum, and mammographic density (Hoffman et al., 2012) in addition to routine screening methods.

## Origins of Breast Cancer

Breast cancer commonly originates in the epithelial layer of the terminal duct system (Fabian et al., 2000). It is unclear exactly how breast cancer begins or what biologic, genetic, or environmental triggers must be present to enable its growth. Most breast cancers are thought to develop in a rather slow fashion with progressive abnormal cellular changes, indicating a multistep and multipath process (Cazzaniga, Decensi, Bonanni, Luini, & Gentilini, 2009). Incidental tissue findings of atypia or lobular carcinoma in situ are recognized as biomarkers that may predict a significant increased risk of developing breast cancer; coupled with a positive family history, a woman’s individual risk may double (Tejada-Berges, 2011). The identification of cellular components that are representative of individual risk factors is tantamount to improved comprehension of breast carcinogenesis and a personalized approach to breast surveillance in high-risk women (Watson & Egland, 2010).

## Methods of Obtaining Breast Cellular Material

Over the past decades, several minimally invasive techniques to obtain breast cellular material have been researched, such as mammary ductoscopy, nipple fluid aspiration, ductal lavage, and random periareolar fine-needle aspiration (RPFNA; Cazzaniga et al., 2009). Mammary ductoscopy utilizes a submillimeter fiberoptic tube to directly visualize the endomammary surface of the duct(s) in the nipple. The primary role of mammary ductoscopy is to determine the pathologic etiology of bloody or nonbloody nipple aspirate, although it may provide a tool to obtain breast cellular material in high-risk women (Mokbel, Escobar, & Matsunaga, 2005).

The nipple fluid aspirate is obtained in several ways, most typically with a commercial unit. The aspirate is obtained from the duct openings following gentle massage or suction, either manually or with a machine. Each breast may or may not produce discernible amounts of nipple fluid aspirate. Samples are placed in a fixative and sent to cytology for identification of atypical cellular components.

Ductal lavage combines manual compression to identify ductal openings that exhibit release of fluid with subsequent cannulation of each fluid-bearing duct. Once cannulated, the duct is flushed with normal saline and aspirated. The withdrawn fluid is placed in a fixative and sent to cytology for identification of atypical cellular components (Hoffman et al., 2012).

## Random Periareolar Fine-Needle Aspiration

Random periareolar fine-needle aspiration is a novel technique that provides a reproducible measure of breast cytology (Hoffman et al., 2012; Ibarra-Drendall et al., 2009; Zalles et al., 2005). This research technique was developed to (1) assess short-term risk of breast cancer development in women and (2) track cytologic response to risk-reduction strategies (Fabian et al., 2000; Hoffman et al., 2012). Random periareolar fine-needle aspiration provides a cellular "snapshot" of a woman’s breast tissue. When four to five passes were taken per site on the breast, 94% of women offered adequate cytology for morphologic assessment (Fabian et al., 2000).

Random periareolar fine-needle aspiration yields a cytologic "field effect" of the breast(s), as cells are obtained from dense parenchymal tissue in the periareolar area. Tissue is deeply surveyed to sample the terminal duct unit, as this is the origin of most breast cancers (Fabian et al., 2000). Cellular material obtained includes epithelial, stromal, adipose, and immune cells. Multiple studies validating the ability to predict short-term (5-year) breast cancer occurrence, reproducibility, and proteomics data (Table 1) have been completed.

**Table 1:**
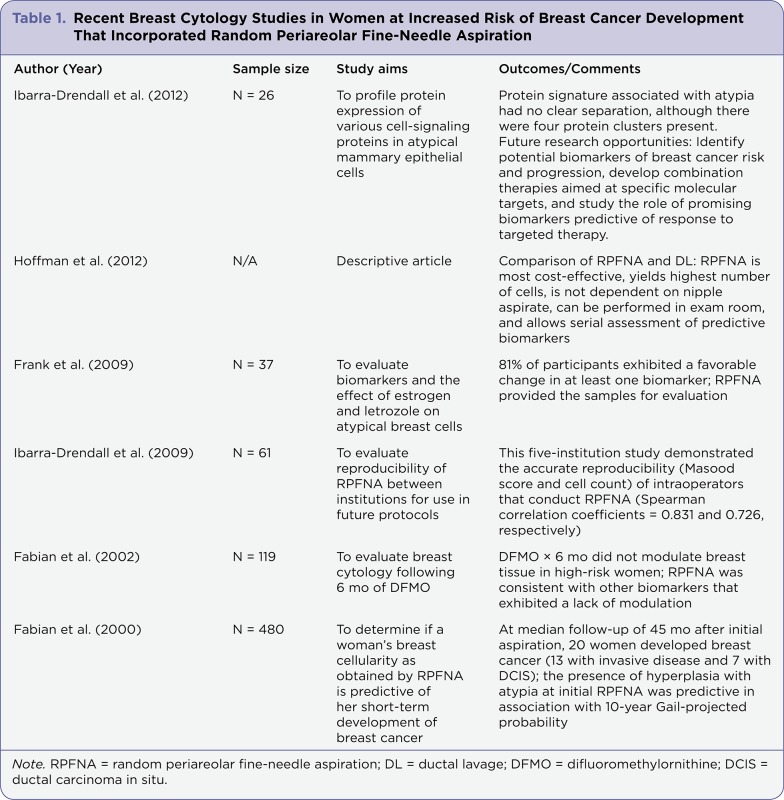
Table 1.Recent Breast Cytology Studies in Women at Increased Risk of Breast Cancer Development That Incorporated Random Periareolar Fine-Needle Aspiration

The RPFNA procedure is quite different from a fine-needle aspiration of a clinically identifiable breast mass, where tissue is obtained from a specific nodule and a pathologic diagnosis of breast cancer is sought (Hoffman et al., 2012). Fine-needle aspiration of a solid malignant tumor can be very painful due to penetration and pressure on a hard tumor, typically without local anesthesia. Women often complain about this painful, albeit brief, procedure. On the contrary, RPFNA obtains random samples from the anesthetized soft tissue of the breast without pressure against a solid barrier. In the authors’ experience, many women repeat the procedure annually, suggesting that RPFNA is well tolerated.

## The Procedure

Random periareolar fine-needle aspiration is generally performed in an outpatient setting by a trained physician, physician assistant, or nurse practitioner. Women are typically enrolled in a formal research study that incorporates RPFNA as a biologic measure of breast cancer risk or as a measure of response to preventive treatment. After verification of a normal breast examination and the presence of dense breast tissue, ice is applied to the upper inner and outer quadrants of the breasts for 20 minutes. This ice application serves to numb the superficial tissue and reduce small-vessel bleeding and resultant hematoma formation during the procedure. After the skin is cleansed with Betadine or chlorhexidine swabs, the overlying skin is anesthetized with approximately 1 cc of 1% lidocaine in a superficial manner. The 25G needle is then inserted more deeply into the breast tissue and an additional 5 cc of lidocaine is administered in a fanlike pattern over 3 to 4 cm of breast tissue, in each upper quadrant, superior to the areola.

Between 12 and 16 10-cc syringes with 21G needles that have previously been primed with sterile saline are used to aspirate samples from each of the 4 quadrants, using 3 to 4 syringes per quadrant. With gentle suction on the syringe plunger, an aspirate of 0.5 to 1 cc of cells is obtained per syringe. This material may contain epithelial, stromal, adipose, and immune cells (Hoffman et al., 2012). It is rinsed in a 12-cc tube filled with 10 cc of a modified CytoLyt solution. The tubes may be separated and marked as "right" and "left" or pooled together in one tube, depending on the study requirements.

Following the procedure, ice and pressure are applied to each quadrant for 20 minutes. Postprocedure, patients are instructed to anticipate superficial bruising, apply ice to the sites on the day of the procedure, wear a snug sports bra, and avoid any activities that cause significant movement of the breasts.

## Interpretation of the RPFNA Sample

The RPFNA material is read by an expert cytologist and evaluated by the Masood cytology index score, which was identified specifically for breast tissue. This score is based on six individual descriptors: cellular arrangement, cellular pleomorphism, myoepithelial cells, anisonucleosis, nucleoli, and chromatin clumping. A sample receives a score of 1 to 4 in each of these categories, for a total score that ranges from 6 to 24. Total scores from 6 to 8 indicate nonproliferative breast disease, 9 to 14 indicates proliferative breast disease without atypia, 15 to 17 indicates proliferative breast disease with atypia, and 18 to 24 indicates carcinoma in situ and invasive cancer. Based on the individual’s Masood scores, women are eligible for a repeat RPFNA either at a 6-month or 1-year interval. If a patient receives a score of 14 or below, she can return in 1 year; if her score is 15 or above, a 6-month repeat RPFNA is recommended to determine the trend.

## Summary

Random periareolar fine-needle aspiration is a novel technique that continues to gain scientific credence in the short-term identification of women at increased risk for breast cancer, or the measurement of interventions designed to decrease risks related to breast cancer. As this technique becomes more widely used, advanced practitioners may seek to be trained in an effort to expand clinical trials, and someday provide a "Pap smear of the breast" for these women who need it most.
